# Deciphering the Physiological Responses to the Intake of Plant-Based Meat Analogues: On the Track of Microbiota and Biomarkers in Serum and Urine

**DOI:** 10.1021/acs.jafc.5c02799

**Published:** 2025-07-17

**Authors:** Guadalupe Sánchez-Terrón, Remigio Martínez, David Morcuende, Mario Estévez

**Affiliations:** 1 TECAL Research Group, Meat and Meat Products Research Institute (IPROCAR), 16759 Universidad de Extremadura (UEX), Cáceres 10003, Spain; 2 Animal Health Department, Animal Health and Zoonoses Research Group (GISAZ), UIC Zoonosis and Emergent Diseases (ENZOEM Competitive Research Unit), 16735 Universidad of Córdoba (UCO, ROR-ID 05yc77b46), Córdoba 14014, Spain

**Keywords:** seitan, tofu, beef, oxidized proteins, metagenomic, metabolomic

## Abstract

Growing concerns about the alleged negative outcomes of the consumption of animal-based foods (ABF) lead a number of consumers to demand so-called plant-based meat analogues (PBMAs), which are designed to replicate the sensory and nutritional characteristics of genuine meat. This study aimed to characterize the physiological effects of long-term PBMA consumption by comparing their specific influence on both gut microbiota and fluids (i.e., plasma and urine) with those of conventional beef in a rodent model by using metagenomic and metabolomic tools. Twenty-one Wistar rats were divided into groups and consumed diets made with either wheat- and soy-based meat analogues or beef. The microbiota of PBMA-fed animals showed increased amounts of the harmful genus of gut bacteria, while their metabolomes reflected disturbances in the nitrogen-containing compound metabolism and the presence of potentially harmful metabolites. These results should raise concern and prompt further research into the long-term impact of consumption of PBMAs on health.

## Graphical abstract

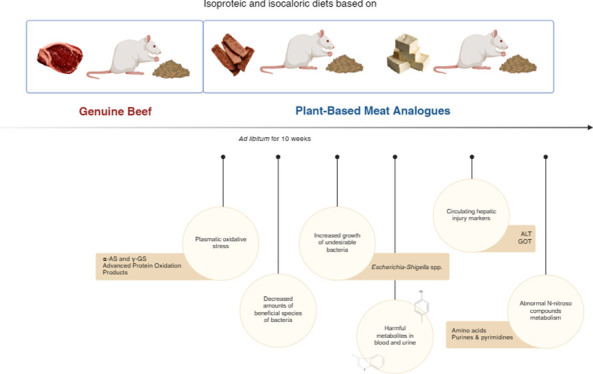

## 1 Introduction

A sheer number of allegations have been made against the consumption of red and processed meat for its plausible association with several gastrointestinal disorders, including the onset and development of colorectal cancer (CRC).^1^ The alleged negative outcomes of meat consumption, together with the increasing awareness of consumers toward animal welfare and environmental protection, are among the reasons why some consumers decide to exclude meat from their diets.^2^ In replacement of meat and other animal-based foods (ABF), some vegan/vegetarians are inclined to include in their diets the so-called plant-based analogues of ABF, which aim to simulate meat appearance and texture. To enhance the interest of these analogues among potential consumers, some of these commercial products are identified as traditional soy and wheat foods, such as seitan or tofu. Yet, it is well-known that some of the industrial tofu and seitan produced and sold in Western countries differ from the traditional Asian products referred to by the same name.^3^ So, these wheat- and soy-based foods may not only be trying to imitate ABF but also traditional seitan and tofu.^3^ It is important to emphasize that while in Western countries this wheat- and soy-based foods are aimed to replace ABF in vegan/vegetarian diets, in Asian countries, these traditional foods are commonly consumed along with meat, chicken, and other ABF. From the Western perspective, these so-called plant-based meat analogues (PBMAs), meat analogues (MA), or vegan meat (VM)^4,5^ are formulated with a number of additives and ingredients and subjected to multiple industrial processing to increase acceptability among consumers. This processing leads PBMAs to be classified as ultraprocessed foods (UPFs), according to the NOVA classification proposed by Monteiro et al.^6^ This system of food classification has recently emerged as a controversial issue due to the exclusion of the final nutritional profile of processed and ultraprocessed foods independently of additives or processing, which should be taken into account when foods are assigned to specific NOVA categories.^7^ While PBMAs are commonly ascribed to positive health outcomes, scientific literature shows gaps of knowledge concerning the actual nutritional value and health impact of these UPFs. The rise in consumption of UPFs is a source of concern due to the noxious effects described by several authors, including overweight, obesity, cardiometabolic risk, type 2 diabetes mellitus (T2DM), cardiovascular diseases (CVD), and cancer.^8,9^ Thus, the World Health Organization (WHO) released a new factsheet in 2021 in which these vegan UPFs were identified as potentially harmful when consumed in the long term as substitutes of ABF.^10^ The crosstalk between diet, microbiota, and health outcomes is a scientific topic of increasing interest.^11–14^ Notwithstanding the knowledge about the impact of UPF consumption, little or nothing is known about the long-term consequences of the UPFs derived from plant material consumption on digestibility, microbiota, and overall health. According to previous studies, the consumption of oxidized proteins from UPFs leads to detrimental health effects on the gastrointestinal tract (GIT)^15^ and on internal organs.^16^ The oxidation-driven chemical and structural changes caused in proteins during food industrial processing could explain the inability of gastric and intestinal peptidases to recognize cleavage sites, thereby leading to impaired digestibility. Such undigested oxidized proteins would remain intact as they pass through to the final sections of the GIT (i.e., colon), where they are causative of dysbiosis and abnormal fermentative events.^15^ Despite these previous findings, a more detailed analysis of the molecular basis of the pathophysiological effects of oxidized proteins from UPFs is required, especially given the involvement of microbiota and chronic inflammatory and oxidative processes in the onset and/or progression of severe intestinal diseases of increasing prevalence today such as CRC.^17^

Therefore, the present study aimed to deeply characterize the shifts in microbial populations at both genus and species levels in Wistar rats fed *ad libitum* with either beef (B), commercial Western-style seitan (S), or commercial Western-style tofu (T), which would be considered processed or UPFs according to several authors,^7^ for 10 weeks using metagenomic analyses. Understanding the molecular basis of physiological responses to the sustained consumption of ultraprocessed vegan foods is crucial to unveil the potential health outcomes of such intake. To fulfill this objective, metabolomic profiling of blood and urine from experimental animals was also carried out.

## 2 Materials and Methods

### 2.1 Chemicals

The reagents used in metagenomic analyses were provided by Thermo Fisher Scientific (Austin, TX, USA). Ultrapure water was prepared using a Milli-Q water purification system (Millipore Corp., Bedford, MA, USA). All other chemicals, solvents, and reagents employed in the analysis were acquired from Sigma-Aldrich (St. Louis, USA) and Fisher Scientific (Hampton, NH, USA).

### 2.2 Meat and PBMAs

Beef loin (M. longissimus dorsi) from industrial genotypes (Charolaise × Limousine) slaughtered at 12 months of age was obtained from a slaughterhouse in Cáceres (Spain) and roasted at 220 °C for 10 min for feed formulation. Samples were collected and aged for 2 weeks at 4 °C before being processed for the manufacture of the experimental feeds. Commercial seitan produced from wheat gluten and commercial tofu were obtained from a renowned supermarket chain in Spain (Mercadona) and submitted to the same cooking procedure prior to their incorporation to the experimental feeds. A more detailed information on ingredients and nutritional composition of both PBMAs is included in the Supporting Information as Table S1.

### 2.3 Animals and Feeds

Twenty-one male Wistar rats were used in our study complied with the Helsinki declaration and the applicable Spanish legal requirements (RD 53/2013), Bioethics Committee of the University of Extremadura (137–2020), and with the approval of the Animal Experimentation Ethical board (EXP20200904) (Regional Government of Extremadura). The animals were supplied by the Animal Experimentation Laboratory at the University of Extremadura (Caceres, Spain). The experimental diets of the rats were elaborated using the rodent’s feed *Teklad Global Diet 2014*, provided by *ENVIGO* (Madison, WI, USA) as a basis. The formulation of the chow was described in detail in our previous work.^15^ The different experimental feeds were developed with the aims of increasing the protein content of the basic formulation (14%) to 30% in the experimental diets and providing isocaloric and isoproteic chows formulated with animal- vs plant-proteins. The different high-protein diets were formulated as follows. The basic feed was milled and then mixed with the exact required amounts of cooked beef, seitan, and tofu to reach the target protein concentration in the final feed. To guarantee the homogeneous mixture of the basal feed with the protein sources, 200 g of water was added every 1000 g of feed. Once minced and mechanically blended for 15 min, the mixture was molded to obtain experimental feed pellets (3 cm length × 1 cm width × 1 m height). The experimental pellets were dried under forced air at 25 °C for 48 h until the moisture content was reduced to approximately 7%. The composition of the experimental feeds was analyzed using official AOAC methods,^18^ and it is showed in Table S2 of the Supporting Information.

### 2.4 Experimental Design

At the beginning of the study, the rats were individually identified through a perforation code in the auditory pavilion. They were maintained in ventilated cages for a week of adaptation during which water and feed were supplied *ad libitum*, and the environmental conditions were controlled (20–22 °C temperature, 40–50% humidity, and 12–12 h light/dark cycle). After this adaptation period, animals were randomly allocated into one of these experimental groups: (i) seitan group (S), which consumed the seitan-based chow (*n* = 7); (ii) tofu group (T), which were fed the tofu-based chow (*n* = 7), and (iii) a control group (B), which received the feed made from beef (*n* = 7). The rationale of these groups is supported by the already formulated hypothesis that commercial PBMAs may lead to different physiological responses than genuine ABF (beef). The experiment was conducted for 10 weeks. Both feed and drinking water were *ad libitum* provided for all groups until slaughter.

### 2.5 Collection of Data on Live Animals

Animals were checked daily to ensure their well-being and safety. During the experiment, food and water consumption was gravimetrically monitored every time they were filled, depending on the demand of the animals (every 2 or 3 days, approximately). Furthermore, body weights were registered weekly.

### 2.6 Euthanasia, Necropsy, and Sampling

At the end of the experimental period, the rats were euthanized by exsanguination via cardiac puncture under 5% isoflurane at an approximate age of 16–17 weeks and an average weight of 437 g. All the blood was collected in tubes with EDTA and subsequently stored at −80 °C. The urine was aseptically collected through puncture of the bladder and properly stored. Feces from the rectum were aseptically collected and stored at −80 °C until metagenomic analyses were performed.

### 2.7 Analytical Procedures

#### 2.7.1 Protein Carbonylation and Pentosidine

The accumulation of protein carbonyls and pentosidine in samples (i.e., feeds and blood) was measured by a previously described method^19^ with slight modifications. HPLC analysis attached to a fluorescence detector was used to quantify specific protein carbonyls (i.e., α-aminoadipic and γ-glutamic semialdehydes (α-AS and γ-GS, respectively)). The samples were homogenized with sodium phosphate buffer solution (PBS), and the remaining steps of the procedure were exactly as those exposed by the above-mentioned authors.^19^ Results were expressed as nmol carbonyl/mg protein, with total primary carbonyls (PPC) being the sum of both semialdehydes. Regarding pentosidine, the results were expressed as fluorescent units.

#### 2.7.2 Advanced Protein Oxidation Products (APOPs)

The analysis of APOPs was carried out using fluorescence spectroscopy (PerkinElmer, Beaconsfield, UK) as reported.^20^ Homogenized samples were diluted with 100 mM sodium phosphate buffer, pH 7.4 with 2 M guanidine chlorhydrate. APOPs were excited at 350 nm, and the emitted fluorescence was recorded from 400 to 500 nm. The excitation and emission slits were both set to 10 nm, and the scanning speed was 500 nm/min. The fluorescence results were applied to a correction factor (Cf = Pt/Pp), where Pt is the total average of the amount of protein from all samples and Pp is the content of protein in each sample. Results are expressed as arbitrary fluorescence intensity (area units) (FU).

#### 2.7.3 Thiobarbituric Acid Reactive Substances (TBARs)

Malondialdehyde (MDA) and other reactive substances were extracted from blood and subsequently quantified as several authors described previously.^21^ Samples from extracts were treated with 8 volumes of perchloric acid (3.86%) and 0.5 volume of butylated hydroxytoluene (BHT) (4.2% in ethanol) to avoid further peroxidation. Samples were placed in a boiling water bath (100 °C) for 45 min together with the tubes from the standard curve (prepared using 1,1,3,3-tetraethoxypropane (TEP) solution in 3.86% perchloric acid) upon reaction with 0.02 M thiobarbituric acid (TBA). After the mixture was cooled, the absorbance was measured at 532 nm by spectrophotometry (Shimadzu Model UV-1800, Shimadzu, Japan). Results were calculated as milligrams of MDA per 100 g of the sample.

#### 2.7.4 Analysis of the Biochemical Profile of Plasma

The stored plasma was sent under suitable conditions to the Internal Medicine Laboratory of the Veterinary Clinic Hospital at the Faculty of Veterinary (University of Extremadura) to obtain a complete biochemical profile of the samples. Specifically, total protein content (TP), albumin (ALB), globulins (GLB), creatinine (CREAT), urea, phosphorus, alkaline phosphatase (ALP), alanine transaminase (ALT), aspartate transaminase/glutamic oxaloacetic transaminase (AST/GOT), triglycerides (TG), total cholesterol, LDL, and HDL were determined. The samples were analyzed in a Saturno 100 VetCrony automatic blood chemistry analyzer (Crony Instruments, Rome, Italy).

#### 2.7.5 Untargeted MS-Based Plasma and Urine Metabolomic Analysis

Metabolites from both plasma and urine samples from Wistar rats were extracted and analyzed. Briefly, 150 μL of plasma and urine were mixed with 200 μL of methanol 100% in order to precipitate undesirable compounds. The mixtures were homogenized in a VWR vortex mixer and subsequently centrifuged at 9000*g* and 4 °C for 10 min. The supernatants were placed in new Eppendorf tubes and dried using a centrifugal vacuum concentrator (Gyrozen, Daejeon, Korea). The residues were reconstituted with 75 μL of methanol (70%) and centrifuged. The supernatants were placed into singled Eppendorf tubes using 0.22 μm nylon-filters. Samples were analyzed using a Dionex UltiMate 3000 RSLC system coupled with a Q-Exactive high-resolution mass spectrometer (Thermo Fisher Scientific, San Jose, CA). Analysis conditions, such as column types, phases, injection volume, or run conditions, were similar to those described in previous works carried out by our group.^22^ The routine calibration and optimization of the equipment, together with the metabolite extraction method, set level 2 of the identification levels proposed by the published literature for the identification and characterization of the metabolites.^23^

#### 2.7.6 Metagenomics Analysis

Microbiota from Wistar rats was analyzed from feces obtained at slaughter using metagenomics analyses. DNA was isolated using the MagMAX Microbiota Ultra Nucleic Acid Isolation Kit (Thermo Fisher Scientific, MA) following the manufacturer’s instructions and the KingFisher Flex Instrument (Thermo Fisher Scientific, Waltham, WA). Specific primers for V3 and V4 variable regions of the 16S rRNA gene were used for genomic DNA amplification (forward: 5′-CCTACGGGNGGCWGCAG-3′; reverse: 5′-GACTACHVGGGTATCTAATCC-3′). Once DNA was amplified, sequencing and basic analysis were performed using an Illumina MiSeq platform using the MiSeq Reagent Kit v3 and 300b paired end. QIIME2 v2021.4 was used for the analysis of the generated raw sequence data. Finally, the operational taxonomic units (OTUs) were classified by taxon using the SILVA database (release 138 QIIME) and trained by a scikit-learn classifier using the UNITE (release 8.3) database. The relative abundance of the different OTUs was analyzed at different taxonomic levels.

### 2.8 Statistical Analysis

The statistical analysis of raw data was carried out by applying parametric and nonparametric tests, based on the normality and homoscedasticity of the raw data using the R statistical software (R.4.3.3.) and SPSS version 29.0. *p* values less than 0.05 were considered statistically significant. For microbiota data, the Bonferroni correction was used to account for multiple comparisons. B-, S-, and T-responsive metabolites were assessed in the MetaboAnalyst 5.0 (https://www.metaboanalyst.ca/). Principal component analysis (PCA) as multivariant analysis was used. The differences between the normalized intensities of the metabolites from each group of samples were evaluated by the ANOVA test as the one-factor statistical method to further analyze the impact of the diets on the plasma and urine metabolome of Wistar rats. FDR-adjusted *p* values, calculated using the Benjamini–Hochberg method, were used to correct for multiple testing in the univariate statistical analysis of metabolomic data. BioRender software (https://BioRender.com) was used to create the figures and the heatmap that illustrated the main metabolomic and microbial changes described.

## 3 Results and Discussion

### 3.1 Gut Microbiota Composition Affected by Replacement of Dietary Beef by PBMAs

The results of the gene sequencing revealed a total of 2,227,534 reads obtained from the 21 fecal samples: 837,334 reads belonged to the B group with a mean value of 119,620 reads per sample, whereas 744,802 and 645,398 reads were obtained from S and T groups, respectively, with mean values of 106,400 and 92,199 reads per sample, respectively. From the reads, we identified an overall total of 316 OTUs, which were grouped into 62 families and 152 genera. The nonparametric Kruskal–Wallis test was applied to assess the differential abundance between the different groups of rats. Post hoc analyses were adjusted using the Bonferroni correction, and the results are grouped in Figure 1.

**Figure 1:**
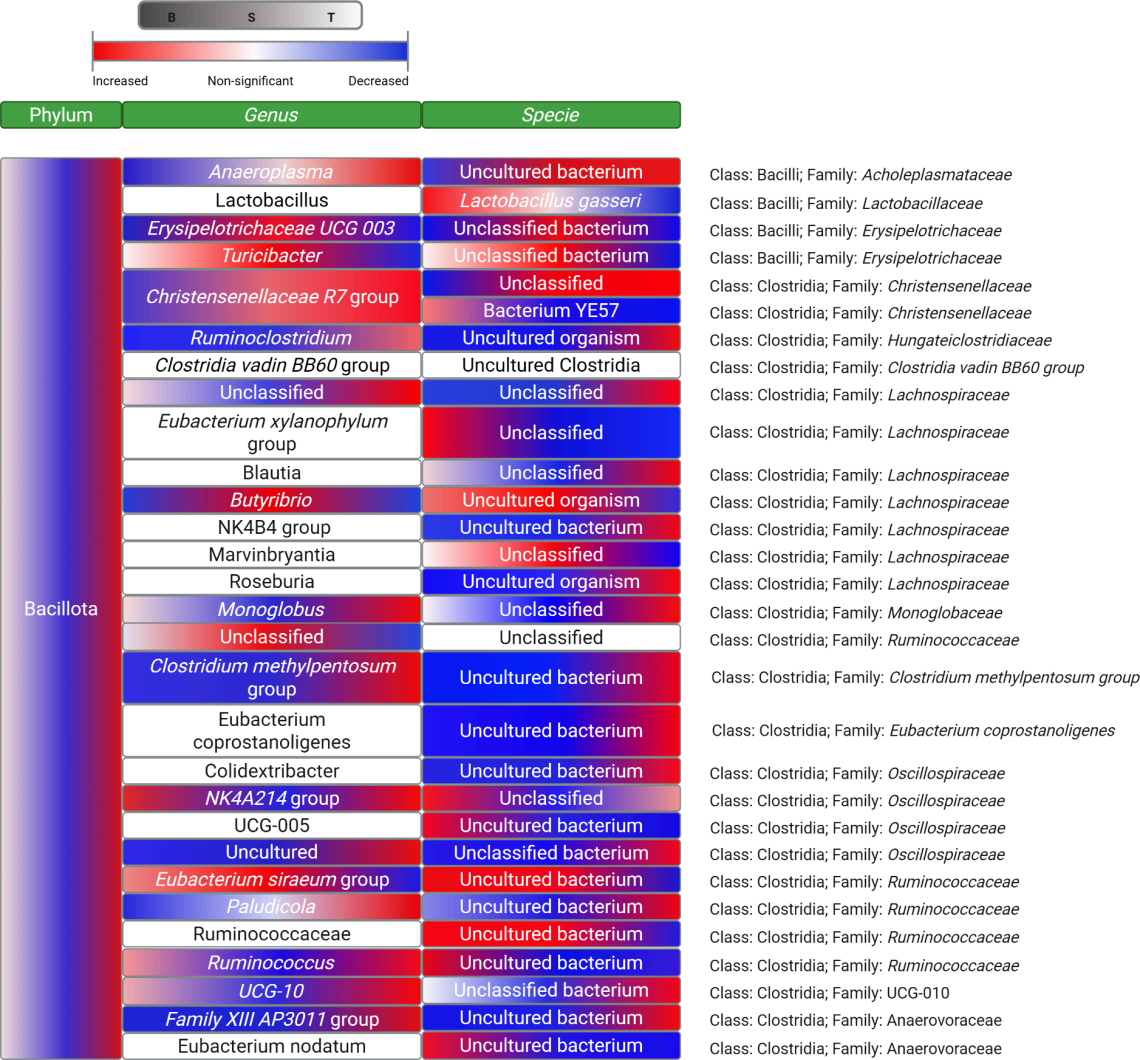
Heatmap illustrating the statistically significant changes in the abundance of microorganisms from feces of Wistar rats fed with beef (B), seitan (S), or tofu (T) at the phylum [i.e., Bacillota], genus, and specie level, respectively. Different colors in the same box mean statistically significant differences in multiple-comparisons posthoc analysis of the relative abundance of each group of microorganisms in the microbiota of B- (left of the box), S- (center of the box), and T- (right of the box) animals (relative abundance value not shown). The trend of the changes is represented in red (increased relative abundance) or blue (decreased relative abundance). *Significant levels in Kruskal–Wallis tests were not specified.

In a previous study, we revealed that different dietary protein sources (i.e., animal- vs plant-based protein sources) affected the microbiota composition of Wistar rats.^15^ Compared to B animals, those fed with PBMAs showed an increased alpha diversity of microbiota in a statistically significant manner, agreeing with previous studies that evaluated the microbiota diversity of humans ascribed to vegetarian vs omnivore diets.^24,25^ However, most available microbiota-related studies assessed whole vegan/vegetarian diets with a lack of information about specific foods consumed by human cohorts. To the best of our knowledge, our study is among novel *in vivo* experiments covering the impact of the sustained consumption of specific commercial PBMAs (regularly included in vegan/vegetarian diets) on the health of experimental animals. Though discussing microbiota data is intricate owing to the complex diversity of microorganisms and biological interactions and the, at times, inconsistent findings from scientific literature, we can state that, in this study, the increased microbial α-diversity in the microbiota of Wistar rats fed on PBMAs was not translated into improved health outcomes. In order to provide further insight into the role played by dietary proteins in gut microbiota, a more detailed discussion of their composition is described as follows.

#### 3.1.1 Bacillota Phylum (Formerly Firmicutes Phylum)

The abundance of Bacillota phyla was also higher in the microbiota of T animals compared to B and S (*p* < 0.05). Conversely, the abundance of the Bacteroidota phylum was lower in the microbiota of rats fed on the T diet as compared to B and S counterparts (*p* < 0.001).

Among the genus and species from the Bacillota phylum, T-microbiota was richer in bacteria such as *Christensenellaceae R7* group spp. (*p* < 0.01), unclassified *Lachnospiraceae* spp. (*p* < 0.05), *Ruminococcus* spp. (*p* < 0.05), or *Paludicola* spp. (*p* < 0.001), among many others (Figure 1). Nevertheless, other uncultured and unclassified species of the Bacillota phylum were found at lower abundance in the microbiota of T rats as compared to their respective B and S counterparts such as *Butyrivibrio* spp. (*p* < 0.05) or *Ruminococcaceae* spp. (*p* < 0.05), among others. In a previous study carried out in Wistar rats, the abundance of uncultured *Lachnospiraceae* spp. was reported to be enhanced in a fructose-induced pro-oxidative and pro-inflammatory gut environment,^26^ agreeing with the current results. Yet, scientific literature indicates that *Lachnospiraceae* spp. (along with others such as *Ruminococcaceae spp.*) are butyrate-producing bacteria that may protect the intestinal epithelium from inflammation.^27,28^ It is worth recalling that the colonic tissue from the rats fed on this soy-based vegan UPF (T) was found to be severely oxidized and inflamed in a previous report.^15^ While the role of *Lachnospiraceae* spp. on human gut health is actually controversial as comprehensively reported by Vacca et al.,^29^ previous studies agree in associating the abundance of this genus with overall poor gut health in experimental animals.^26,30,31^

The other group of bacteria from Bacillota with a recognized impact on gut health are those from the *Lactobacillus* genus. The replacement of B by the PBMAs in the diet of Wistar rats led to a remarkable 6- and 9-fold lower relative abundance of *Lactobacillus gasseri* in S- and T-microbiota, respectively (*p* < 0.05) (Figure 1). Unlike *Lachnospiraceae* spp., the beneficial effects of this species on human gut health are generally recognized. However, the impact of soy-based products on the abundance of these species is not fully consistent in the literature. In some studies, fermented tofu and tempeh were found to enhance the abundance of these lactic acid bacteria in the gastrointestinal tract of experimental animals and human beings with proven health benefits.^32,33^ However, authors such as Zhu et al.^34,35^ reported that animal protein-based diets (meat/fish) promoted the growth of *Lactobacillus* spp. in rats in a significantly higher fashion than soy-based protein diets, which is in agreement with our results. It is documented in scientific literature that *Lactobacillus* is highly sensitive to the concentration and nature of dietary proteins arriving to the large intestine.^36^ In line with the present results, some authors reported a diminished abundance of *Lactobacillus* spp. as a consequence of the intake of oxidized proteins in various murine models.^26,37,38^

#### 3.1.2 Desulfobacterota Phylum

The abundance of *Desulfovibrio* spp. was higher in the microbiota of T rats, followed by the microbiota of B and S counterparts (*p* < 0.05) (Figure 2). In fact, the OTUs identified as *Desulfovibrio fairfieldensis* were 2-fold and 3-fold higher in the microbiota of T rats than in those from B and S animals, respectively (*p* < 0.05). While this group of microorganisms was not among the dominant, the shift observed between diets was of biological significance.

**Figure 2:**
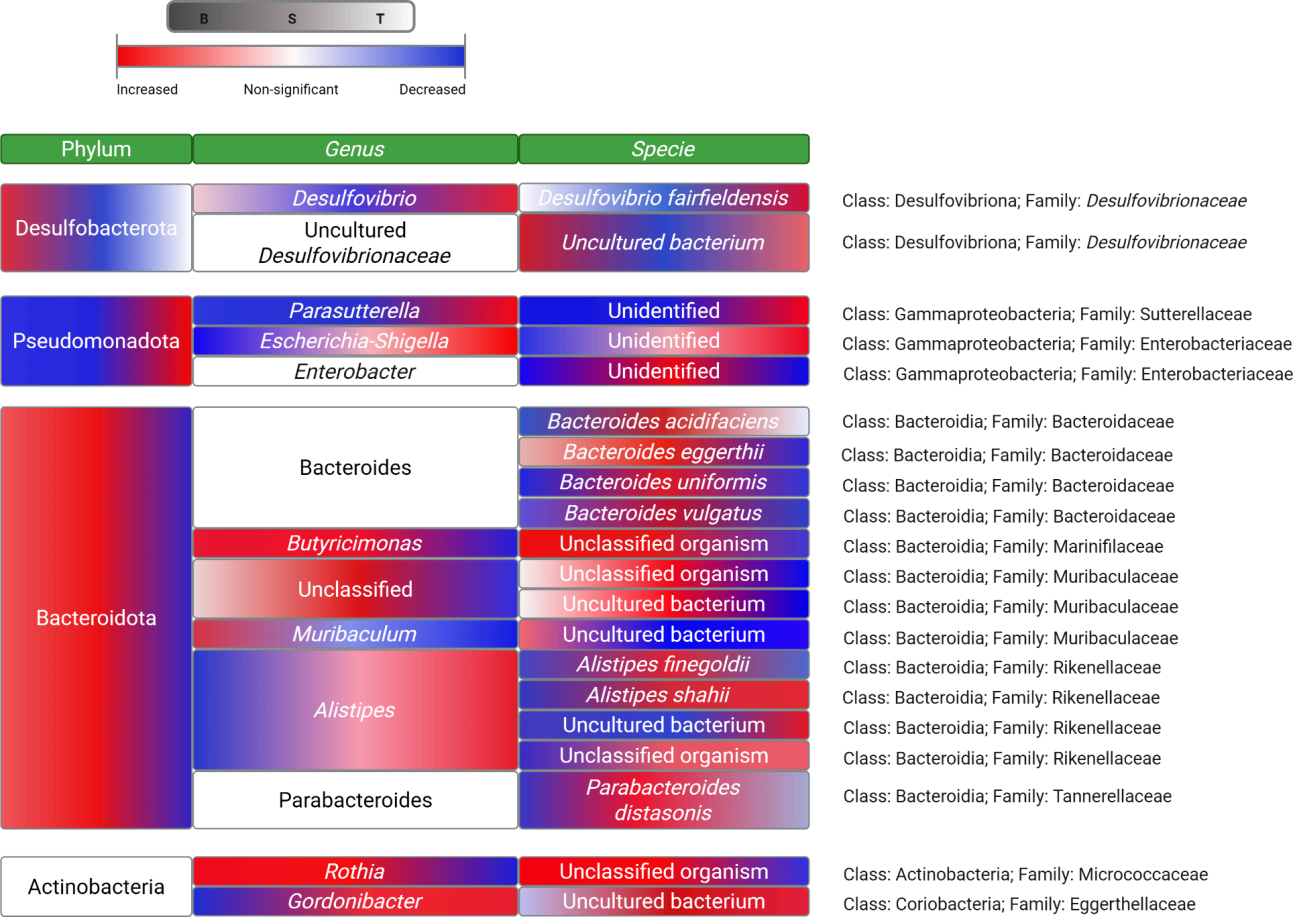
Heatmap illustrating the statistically significant changes in the abundance of microorganisms from feces of Wistar rats fed with beef (B), seitan (S), or tofu (T) at the phylum [i.e.: Desulfobacterota, Pseudomonadota, Bacteroidota, and Actinobacteria], genus, and specie level, respectively. Different colors in the same box mean statistically significant differences in multiple-comparisons posthoc analysis of the relative abundance of each group of microorganisms in the microbiota of B- (left of the box), S- (center of the box), and T- (right of the box) animals (relative abundance value not shown). The trend of the changes is represented in red (increased relative abundance) or blue (decreased relative abundance). *Significant levels in Kruskal–Wallis tests were not specified.

Sulfate-reducing bacteria (SRB) are part of the normal gut microbiota, but increased levels of this group of microorganisms may contribute to the development of intestinal bowel diseases (IBD), likely linked to the production of hydrogen sulfide (H_2_S) and related-metabolites. SRB reduce sulfates, sulfites, and sulfated polysaccharides derived from both dietary amino acids and local mucins, negatively affecting the gut environment, intestinal mucus barrier integrity, the liver, and other peripheral organs.^27,39,40^ In contrast with our results, most studies have reported a lower abundance of *Desulfovibrionaceae* in the microbiota of human vegan/vegetarians compared to omnivorous individuals.^24^ However, these results should be compared with caution to our results due to the different experimental conditions. Nakamura et al.^41^ identified the phylum Desulfobacterota as dominant in mice fed a low vs high protein soy-based diet (5% vs 20%) for 6 weeks. The authors, who related this phylum to inflammatory events, attributed the undesirable changes in the microbiota of animals to protein deficiency. Similar results were found in this study for Wistar rats fed the soy-based PBMAs, while, in this case, isoprotein diets were provided to all animals. Yet, the results from both studies may be attributed to similar mechanisms. We hypothesize that, further to the amount of dietary protein, chemical and structural alterations in proteins induced during ultraprocessing of PBMAs may play a crucial key role in the digestibility of such proteins, their actual nutritional value, and eventually in microbiota composition. In fact, markers of protein oxidation displayed on Table 1 show that the soy-based PBMA supplied to the animals had significantly higher concentration of oxidized and aggregated proteins compared to the feeds elaborated with wheat gluten (S) and beef (B) (*p* < 0.001). Additionally, in a previous study, we found that such oxidized proteins from T were significantly less digestible than S and B proteins in the stomach and jejunum of Wistar rats.^15^ Other authors found consistent results when assessing the impact of severe processing on the extent of protein aggregation and decreased digestibility in PBMAs compared to real meat.^42^ Therefore, the actual delivery of essential amino acids may not only depend on the load of dietary proteins but also on their effective digestion in the GIT. Hence, the “protein deficiency” reported by Nakamura et al.^41^ may apply to the present situation in which highly oxidized proteins from a soy-based UPF would lead to an impaired protein supply as compared to other more digestible dietary proteins. Estévez and Xiong^43^ hypothesized that undigested oxidized and severely aggregated proteins from processed foods may reach the colon, promoting oxidative stress in the gut environment and dysbiosis. Recent studies have proven that this hypothesis is plausible. In a first study, fructose-induced oxidation of dietary proteins led to reduced digestibility, increased gut fermentation, colonic oxidative stress, and dysbiosis in Wistar rats.^26^ More in particular, increased abundances of *Desulfovibrio* spp. were reported when undigested oxidized proteins reached the colon. Consistent results were obtained by Yin et al.^38^ when assessed the impact of the intake of oxidized meat proteins in mice. Since evidence suggests that *Desulfovibrio* spp. and colonic H_2_S production lead to DNA damage partly due to reactive oxygen species (ROS) generation,^44^ we must take into consideration the negative impact of the consumption of oxidized proteins from UPFs on microbiota and extensively on health, irrespective of whether such dietary proteins were originally obtained from animal or plant sources.

**Table 1:** Concentrations of Markers of Oxidative Stress (Means ± Standard Deviation) in the Different Isoprotein and Isocaloric Experimental Feeds^a^ Differing in Sources of Protein Supplementation (Beef (B), Commercial Seitan (S), or Commercial Tofu (T))

marker	B	S	T	
*p* value^b^				
α-AS (nmol carbonyls/mg protein)	0.42c ± 0.11	1.02b ± 0.23	1.56a ± 0.25	***
γ-GS (nmol carbonyls/mg protein)	0.21c ± 0.04	0.45b ± 0.08	0.69a ± 0.11	***
total PPCs (nmol carbonyls/mg protein)	0.64c ± 0.07	1.48b ± 0.23	2.25a ± 0.32	***
APOPs (nmol carbonyls/mg protein)	178c ± 40	389b ± 49	498a ± 53	***
TBARs (nmol carbonyls/mg protein)	0.06c ± 0.01	0.10b ± 0.01	0.36a ± 0.02	***

a Experimental feed as explained Materials and Methods.

b Significance level in ANOVA; *p* < 0.05; **: *p* < 0.01; ***: *p* < 0.001; ns: no significant. Means with different letters within the same row were significantly different in Tukey posthoc analyses.

#### 3.1.3 Pseudomonadota Phyla (Previously Classified as Proteobacteria Phylum)

The almost 30-fold higher abundance of *Escherichia-Shigella* spp. in the microbiota of T animals compared to those from the B group (*p* < 0.05) is highly remarkable. On the same line, 9-fold higher abundance of those genus of bacteria was found in the microbiota of S Wistar rats than in that from the B counterparts (*p* > 0.05). The relative abundance of an unidentified bacterium from the *Enterobacter* genus was also significantly higher in the microbiota of S rats compared to their B and T counterparts (*p* < 0.01). Moreover, an unidentified species of *Parasutterella* spp. was shown to be increased in the microbiota of T and S animals compared to B (*p* < 0.05 and *p* > 0.05, respectively), with animals from the T group having the highest abundance of this bacteria among those eating the PBMA-based diets. The differential growth of bacteria from the Enterobacteriaceae family in the gut has been linked to the fermentation of undigested proteins and represents a risk factor for IBD and CRC.^40^ The intake of animal-based foods rich in fat and proteins, as well as the intake of oxidized proteins from animal-based UPFs, has been previously associated with a dominant position of genera *Escherichia-Shigella* in the microbiota of experimental animals.^25,45^ However, to the best of our knowledge, no previous studies have addressed the possibility that increased amounts of *Escherichia-Shigella* spp. and related Enterobacteriaceae genera are associated with the consumption of ultraprocessed PBMAs. Sidhu et al.^24^ reported contradictory results from scientific literature when describing the role of the Enterobacteriaceae family in the microbiota of individuals having a vegan/vegetarian diet as compared to the microbiota of omnivorous subjects. Zhou et al.^46^ showed increased counts of the *Citrobacter* genus in a simulated microbiota when the replicated gut environment was exposed to plant-derived burger patties vs beef-based products. Although differences in the nature of the models and the food under study hinder a straightforward comparison between studies, it seems reasonable that not only the load of dietary proteins but also its nature and extent of processing in relation to its oxidative status, in particular, could explain shifts in microbiota composition. The data from previous studies and the current ones, taken together, support the hypothesis that the consumption of high amounts of oxidized proteins, regardless of their source, contributes to the development of potentially pathogenic bacteria in the gut, associated with a high prevalence of chronic intestinal diseases. Hence, it seems advisible that whenever vegan/vegetarian diets are to be assessed in relation to microbiota composition and health outcomes, the extent of processing of the foods from such diets may be taken into account. The design and intake of a vegan diet based on nonprocessed/minimally processed plant-sourced foods may plausibly have different outcomes than a diet in which vegan UPFs are regularly included.

#### 3.1.4 Bacteroidota Phylum

The microbiota of B and S rats had similar abundance of the genus *Butyricimonas*, while that from the T animals had a significantly lower abundance of this group of bacteria (*p* < 0.05, respectively). Some species from the *Butyricimonas* genus have been previously linked to relief from glucose-mediated metabolic disturbances associated with obesity.^47^ The authors of this previous study showed that several intestinal mRNA transcripts, related to the intestinal epithelial barrier, were upregulated by the microorganisms in a murine model. This would provide a stronger basis for hypothesizing that the lower abundance of *Butyricimonas* spp. in the microbiota of T animals might play a role in the colonic disturbances previously shown in the rats.^15^

Moreover, compared to B, the microbiota from T rats also showed significantly lower relative abundance of *Muribaculum* spp. (*p* < 0.01) and significantly higher amounts of *Alistipes* spp. (*p* < 0.01). *Muribaculum* bacteria were described as beneficial microorganisms, since several authors showed significant negative correlations between *Muribaculum* richness and plasma cholesterol.^48^ However, there are studies that reported contradictory results, relating increased abundances of the genus with aggravated lipid metabolism in male mice.^49^ Table 3 shows the statistical analysis of biochemical results from the plasma of experimental rats. Increased amounts of circulating LDL in T rats (*p* < 0.05) could be related to the lower abundance of *Muribaculum* spp. in line with the previous reports, but the highest levels of the lipoprotein were highlighted in the blood of S rats (*p* < 0.05). However, *Muribaculum* spp. in the microbiota of S rats did not show significant differences at the genus level after statistical analysis, respective to T and B counterparts. Regarding *Alistipes* spp., *Alistipes shahii* was found to be more abundant in the microbiota of S compared to B (*p* < 0.01), while no differences in the abundance of the microorganism were found between the microbiota of the animals having the PBMA diets. Also, an unclassified species from the *Alistipes* genus was significantly more abundant in the microbiota of T animals, followed by that from S, while the microbiota from B animals had the lowest abundance of this bacteria (*p* < 0.05). Since *A. shahii* has previously been associated with high rates of luminal colonic oxidative stress,^26^ its increased prevalence in the groups of animals fed with the PBMAs is consistent with the increased values of protein oxidation markers showed in the PBMA feeds (Table 1) and with our previous findings in which the intake of these PBMAs promoted both luminal and tissue oxidative stress in the GIT of Wistar rats.^15^

Other particular species from the Bacteroidota phylum reached the highest abundance in the microbiota of S animals (vs B and T), such as *Bacteroides acidifaciens* (*p* < 0.01), *Bacteroides eggerthii* (*p* < 0.05), *Bacteroides uniformis* (*p* < 0.05), and *Bacteroides vulgatus* (*p* > 0.05) (*Bacteroides* spp.), as well as *Parabacteroides distasonis* (*p* < 0.05) (*Parabacteroides* spp.). Extensive literature reported animal-based diets to be responsible for the increase of microorganisms from the Bacteroidota phylum.^50,51^ However, there is a close relationship between *Bacteroides* and polysaccharides degradation,^52^ so it is not surprising that this genus of bacteria was increased in the microbiota of the animals fed a wheat gluten-based diet (S) with higher levels of carbohydrates than T and B (Table S1). Although the above-mentioned authors attributed beneficial effects to the microorganisms, researchers such as Bascuñán et al.^53^ linked *Bacteroides* spp. with gluten metabolism and celiac disease, which is consistent with the rise of inflammatory markers in the intestine of Wistar rats fed the S diet for 10 weeks.^15^ Moreover, together with *Bacteroides*, increased abundance of the Pseudomonadota phylum was reported to play a role in the adverse effects of the intake of gluten-based foods.^53^ Other studies have also linked an increase in Bacteroidetes and Pseudomonadota phyla, as well as a decrease in Bacillota, to metabolic diseases such as T2DM or insulin resistance.^54^ Therefore, our results should be taken into consideration when replacing meat with meat analogues such as commercial seitan for this potential involvement in intestinal and metabolic disorders, plausibly mediated by shifts in microbiota.

The changes described by microbiota due to different diets may lead to the release of different metabolites into both the colonic environment and the blood as a consequence of the differential microbial use of the foods under study. Moreover, it is reasonable to hypothesize that different metabolites will be transferred to blood and eventually excreted through urine, so an accurate untargeted metabolomic study of the fluids from experimental animals (i.e., blood and urine) will be addressed in the following section.

### 3.2 Metabolomic Profiling of Plasma and Urine Affected by Replacement of Dietary Beef by PBMAs

To further investigate the role played by the microbiota on the generation and fate of metabolites formed during the digestion of the foods under study, blood and urine from B, T, and S Wistar rats were subjected to a nontargeted metabolomic analysis. The assay revealed 1080 metabolites in the plasma from B, S, and T Wistar rats. Compound Discoverer software paired the compounds name and/or formula with the calculated weights of the detected molecules using different databases (i.e., AKos, BioCyc, Chemspace, FooDB, Human Metabolome Database, KEGG, LipidMAPS, Mcule, Nature Chemical Biology, Nature Chemistry, NPAtlas, Toxin, Toxin-Target Database, and Urine Metabolome Database). Overall, 295 metabolites were detected in only the plasma of B rats, 222 were only found in the plasma of S animals, and 202 were only found in the plasma of T rats. Moreover, 372 metabolites from plasma showed statistically different abundances among the groups (ANOVA). On the other hand, 1716 metabolites were detected in the urine from the animals. 500 metabolites were only detected in the urine from B animals, while 473 and 366 were only found in PBMAs (seitan and tofu groups, respectively). Additionally, based on the ANOVA results, 538 metabolites were found at significantly different concentrations in urines from the three experimental groups. To improve the readability of the work and the comprehension of the results, only the most relevant results from the one-factor statistical test are presented and discussed. To analyze the data, the peak intensities of the metabolites were compared using Metaboanalyst software. Figures 3 and 4 show the differential abundance of the most relevant metabolites in both blood and urine, respectively, according to the ANOVA test. For a better comprehension of the results, metabolites are grouped and discussed as affected by common metabolic pathways.

**Figure 3:**
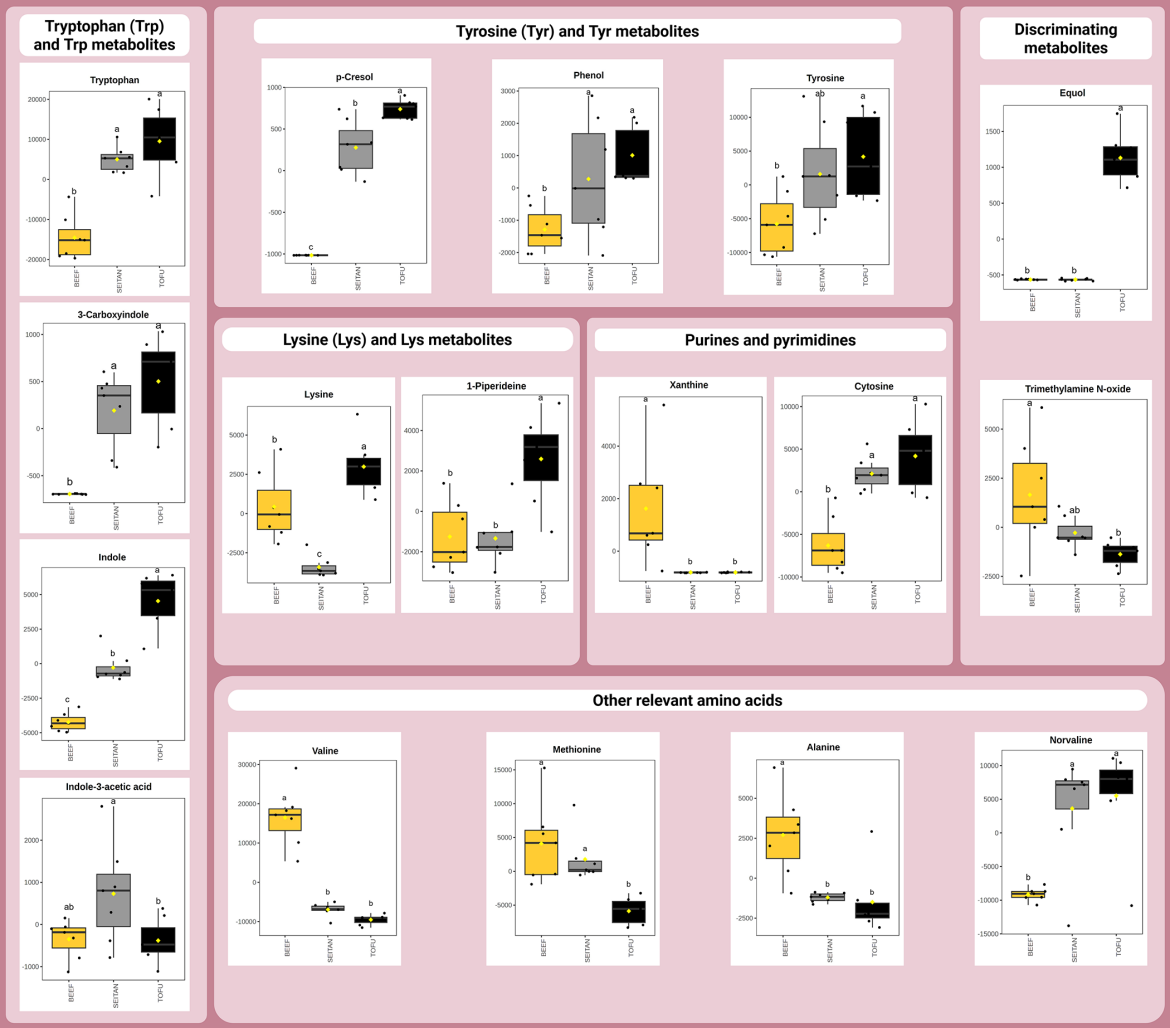
Differential abundance of blood metabolites affected by the different feeds: beef (B), seitan (S), and tofu (T).

**Figure 4:**
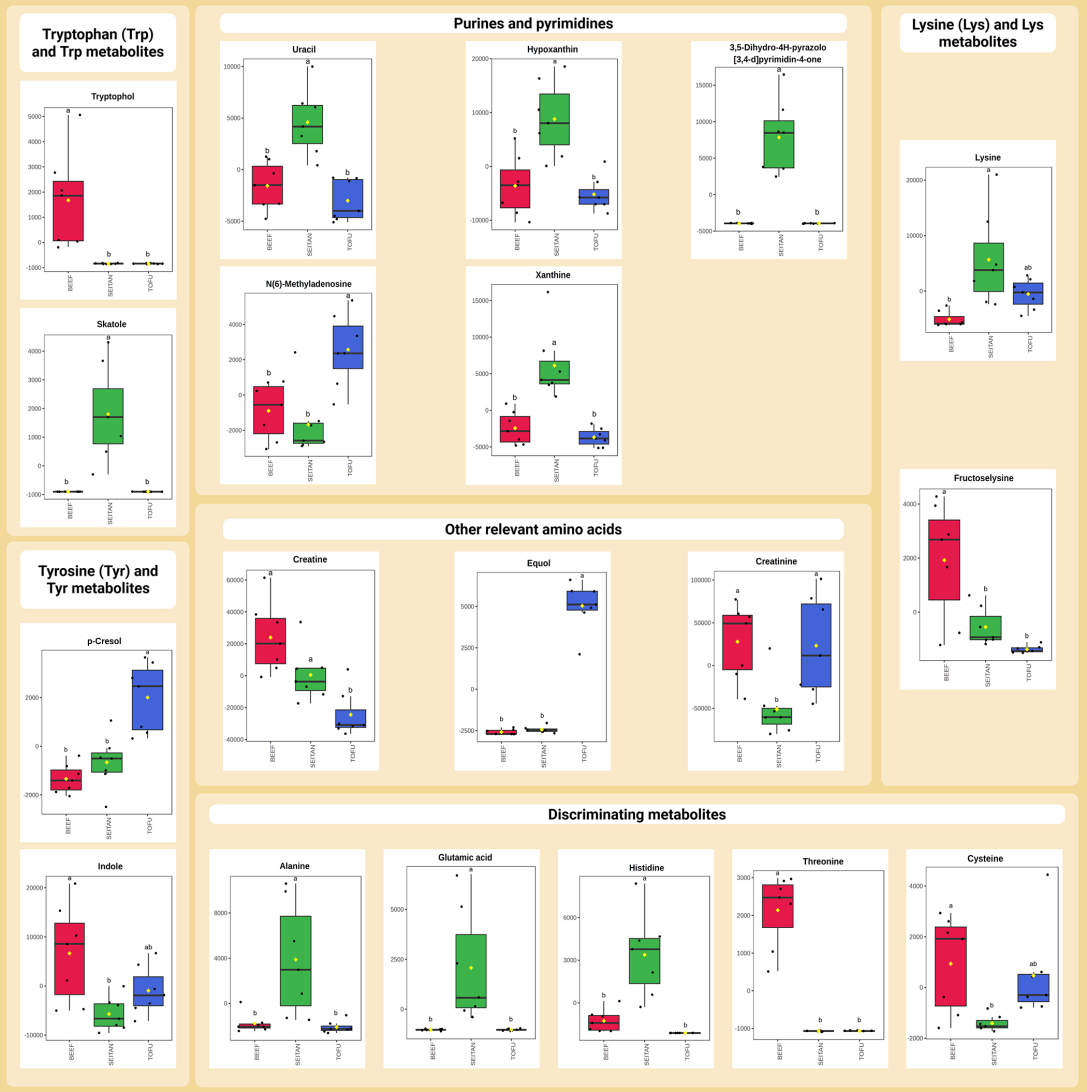
Differential abundance of urine metabolites affected by the different feeds: beef (B), seitan (S), and tofu (T).

#### 3.2.1 Tyrosine (Tyr) and Tyr Metabolites

In a previous study, the untargeted metabolomics of digests from these three experimental diets already showed remarkable differences in relation to amino acid release and metabolism.^15^ Amino acids could be used to build microbial protein, but they can also be fermented as an energy source, resulting in metabolites that affect the host beyond amino acid availability.^55^ Tyrosine is one of those amino acids, and the anaerobic fermentation of Tyr is known to lead to two major metabolites, *p*-cresol and phenol.^56^ In our previous study, we reported that the consumption of diets enriched with PBMAs (namely T) led to the occurrence of *p*-cresol as a characterizing metabolite from the colonic fermentation of T.^15^ Consistently, significantly higher concentrations of *p*-cresol were found in both blood and urine of PBMA groups, especially in the urine of T rats, as compared to fluids from B counterparts (Figures 2 [Fig fig3] and 4). As already reported, around 35% of the dietary T proteins, poorly digested in the stomach and jejunum, arrived at the colon of Wistar rats fed on T.^15^ S was also found to display a shifted digestion pattern as compared to B. This shifted digestion patterns of proteins from PBMAs, plausibly caused by the severe processing and extent of the oxidation of the commercial products used to make the feeds (Table 1), would explain the colonic dysbiosis and the differential metabolomic signature in colon, blood, and urine. Saito et al.^57^ made a thorough identification of *p*-cresol-producing intestinal bacteria. *Clostridium difficile* ^58^ and *Clostridium scatologenes* ^59^ are associated with the last conversion of *p*-cresol from 4-hydroxyphenylaceate in the colon. In the present experiment, an uncultured bacterium of the *Clostridium methylpentosum* group was more abundant in the microbiota of T animals (*p* < 0.001), but it is unknown to what extent other species from the Clostridia class play a role in *p*-cresol formation. Saito et al.^57^ defined that *p*-cresol producing bacteria belonged to *Clostridium* clusters XI and XIVa,^57^ which would make unlikely that the *C. methylpentosum* group was involved in the production of the harmful metabolite, since the genus was affiliated to *Clostridium* cluster IV.^60^ So, given the presence of this compound, there is no doubt that the presence of other bacteria with *p*-hydroxyphenylacetate decarboxylase or tyrosine lyase activity, or both, may have contributed to the production of *p*-cresol, but an in depth study of correlations between species and metabolites would be necessary. While the bacteria implicated in the formation of *p*-cresol in this study remain indefinite, its occurrence in colonic digests, blood, and urine of T rats is a finding of biological significance. *p*-Cresol and its conjugates are profusely documented to display genotoxic effects in a high protein diet context.^58^ Additionally, based on the novelty of the present results, circulating Tyr-microbial metabolites, *p*-cresol and phenol may be associated with the intake of plant-based UPFs in comparison with an animal-based isoprotein diet. As aforementioned, we hypothesize that the arrival of undigested oxidized proteins from T to the colon would explain the fermentation of Tyr residues and, hence, the formation of these metabolites. Both *p*-cresol and phenol are chemical species of recognized toxicological concern as they have mutagenic properties, and both *p*-cresol and its conjugated forms are uremic toxins that correlate positively with chronic kidney disease and diabetes pathogenesis.^61^ Unlike *p*-cresol, traced at high urine concentrations in T rats, phenol was not found as such in animals fed the PBMA, which would indicate accumulation or further reactivity prior to excretion.

#### 3.2.2 Tryptophan (Trp) and Trp Metabolites

In the present study, higher Trp blood concentrations were found in animals fed the PBMA as compared to those fed on B (Figure 3). Circulating Trp is known to play a role in host health since the amino acid is an important precursor of biologically active compounds such as serotonin, melatonin, or coenzymes that are important to redox balance, among others.^58,62^ Thus, Trp is a precursor of modulators of mood and behavior in humans, having antidepressant and anxiolytic actions.^63^ Authors such as Poesen et al.^64^ reported dissimilarities in Trp plasma levels between humans and mice fed high protein diets, showing mice a higher concentration of Trp in plasma. However, to the best of our knowledge, our results are novel, revealing that not only protein concentration but also the source of dietary protein promotes differences in plasma Trp levels in experimental murine models.

Additionally, a differential microbiota metabolism of Trp could explain the higher concentrations of Trp-derived metabolites in the blood of experimental animals fed on the PBMAs compared to those from B rats (Figure 3). Although the majority of Trp derived from ingested proteins is absorbed in the upper GIT, a certain amount of Trp can still reach the large intestine, where it is degraded by a range of commensal microbes.^65^ Current evidence indicates that undigested oxidized proteins from PBMAs reached the colon of T and S rats at higher concentrations than in B animals.^15^ It is hence plausible to hypothesize that the microbial utilization of these aggregated and poorly digested proteins in previous stages of digestion could have provided gut microbiota with higher loads of Trp for microbial catabolism. At this location, Trp could have been catabolized by microbiota mainly along different pathways, and in our study, Trp was mainly degraded into indoles and indole-derivates.^66^ Compared to the blood from B animals, that from rats fed on PBMAs showed the highest abundance of indole (*p* < 0.001; FDR-adjusted *p* < 0.001) and 3-carboxyindole (*p* < 0.001; FDR-adjusted *p* < 0.001). The urine metabolomic profiling also revealed differences in relation to Trp metabolism between diets as animals fed on S showed the highest concentration of skatole (*p* < 0.001; FDR-adjusted *p* < 0.001), while B animals had tryptophol as a characterizing urine Trp metabolite (*p* < 0.01; FDR-adjusted *p* < 0.01). Indoles, including indole, indole 3-acetic acid (IAA), indole-3-propionic acid (IPA), indole-3-aldehyde (IALD), and indole-3-acetaldehyde (IAALD), among others, play an important role in modulating the expression of inflammation-related genes and epithelial cell barrier homeostasis.^67^ The Trp indole pathway seems to be modulated by several microorganisms, such as *Bacteroides* spp. or *Escherichia coli*,^66,67^ which were found in increased abundance in the microbiota of Wistar rats fed the PBMAs, providing strength to the connection between microbiota composition and Trp metabolism. While, at this point, the microbial-mediated formation of Trp-derived indoles may be regarded as an event of potential positive physiological consequences, other Trp metabolites indicate otherwise. In particular, it is important to note the notably higher abundance of skatole in the urine of animals that consumed S (*p* < 0.001; FDR-adjusted *p* < 0.001). Skatole is also derived from the metabolism of Trp by intestinal bacteria, and it is generally accompanied by the production of other deleterious protein-derived metabolites such as *p*-cresol.^68^ Skatole is largely related to gastrointestinal diseases and chronic kidney disorders and may be regarded as a negative outcome of metabolic use that microbiota would make of Trp in animals fed with S, in particular.^68^

#### 3.2.3 Metabolism of Other Relevant Amino Acids

The metabolomic profiling of blood revealed differences in the abundance of free amino acids when different protein-source diets were consumed by Wistar rats for 10 weeks. For example, lysine (Lys), valine (Val), methionine (Met), and alanine (Ala) also showed different abundances in the blood of the animals depending on the dietary treatment (Figure 3). Regarding the quantity of Lys, the blood from T showed higher amounts of this amino acid in contrast with their counterpart groups (*p* < 0.001; FDR-adjusted *p* < 0.001), with S being the group with the lowest abundance of circulating Lys. Conversely, the S animals excreted though urine higher amounts of Lys than T and B, respectively (*p* < 0.01; FDR-adjusted *p* < 0.05) (Figure 4), which may explain their blood fingerprint. Lysine is an indispensable amino acid (IAA) that must be consumed in suitable amounts to maintain protein synthesis.^69^ The higher urinary excretion of lysine observed in S animals could be a hallmark of a differential and plausible defective metabolism of the amino acid apparently promoted by the consumption of different protein sources. Since the liver appears to be the main site of lysine catabolism,^69^ the highest concentration of the enzymatic liver injury markers (i.e., ALP, ALT, and GOT) in blood from S rats (Table 3; *p* < 0.05) would support a liver damage and the abnormal fate of lysine in S animals. In contrast, Lys-related metabolites (i.e., 1-piperideine) were shown in higher quantities in the blood of T animals (Figure 3) than in that of S and B counterparts (*p* < 0.001; FDR-adjusted *p* < 0.01). 1-Piperideine derives from l-lysine catabolism, directly formed from the decarboxylation of lysine-product cadaverine.^70^ 1-Piperideine is an intermediate in the formation of 5-aminopentanoic acid, which is a methylene homologue of γ-aminobutyric acid (GABA) that acts as a weak agonist of GABA,^71^ supporting the differential amino acid metabolism associated with the diets previously hypothesized. Moreover, the urine of B animals showed remarkable increased amounts of fructoselysine (*p* < 0.001), a marker of protein glycation. The urine excretion of this Lys-derived metabolite in B animals (Figure 4) contrasts with the elevated amounts of pentosidine in S and T blood showed in Table 2 (*p* < 0.001). Pentosidine is a marker of advanced protein glycation, and these results suggested, in line with the previously exposed, that the intake of oxidized protein derived from the diets made with commercial PBMA products may have increased the intraluminal oxidative stress and negatively affected gut health and liver function, affecting amino acid metabolism, among other consequences.

**Table 2:** Concentrations of Markers of Oxidative Stress (Means ± Standard Deviation) in the Blood of the Experimental Animals after 10 Weeks of *Ad Libitum* Intake of the Different Experimental Diets

marker	B	S	T	
*p* value^a^				
α-AS (nmol carbonyls/mg protein)	0.38b ± 0.03	1.14a ± 0.18	1.10a ± 0.17	***
γ-GS(nmol carbonyls/mg protein)	0.11c ± 0.02	0.38b ± 0.08	0.62a ± 0.16	***
total PPCs (nmol carbonyls/mg protein)	0.50b ± 0.05	1.53a ± 0.24	1.73a ± 0.26	***
pentosidine (fluorescent units)	0.45c ± 0.07	1.34a ± 0.09	0.80b ± 0.11	***
TBARs (mg MDA/kg sample)	0.54c ± 0.07	1.30b ± 0.10	1.59a ± 0.27	***

a Significance level in ANOVA; *p* < 0.05; **: *p* < 0.01; ***: *p* < 0.001; ns: no significant. Means with different letters within the same row were significantly different in Tukey posthoc analyses.

Moreover, the abundance of Val was lower in the blood from S and T animals than from B rats (*p* < 0.001; FDR-adjusted *p* < 0.001). In this line, authors such as Wang et al.^72^ revealed a close relationship between vegan/vegetarian diets and branched-chain amino acids (BCAAs), showing lower circulating BCAAs in human vegetarians vs. omnivores, which is in line with the results from our mice model. Additionally, the authors showed significantly upregulated gut microbial pathways for the degradation of these BCAAs in vegetarian groups compared to their omnivore counterpart, relating increased amounts of the *Prevotellaceae* family in human vegan/vegetarian groups, among other taxa, with the modulation of circulating BCAAs. Despite not sharing this outcome, the multitude of taxa modified by the PBMA diets compared to the B diet, and the evidence that suggested impairments in the amino acids metabolism, the decreased amounts of circulating BCAA could mean a differential growth of microorganisms and defective use of amino acids when oxidized proteins are present in the food. However, the divergences among the experimental settings and the lack of information about the specific vegan/vegetarian foods included in the diets do not allow a reasonable comparison of the results.

The intake of B diets led to significantly higher blood concentrations of other essential and nonessential amino acids such as Met and Ala as compared to results from rats fed the PBMAs (*p* < 0.001; FDR-adjusted *p* < 0.01 in both cases). Overall, there did not seem to be a correspondence between blood metabolome and urinary amino acid excretion in animal groups, which has been hypothesized to have an important health effect.^73^ Protein fermentation by gut microbiota may contribute to host amino acid balance, generating a diverse range of bioactive molecules, which exert multiple host effects, such as inflammatory response, tissue permeability, and colitis, among others.^55^ Amino acids are the basic unit for protein synthesis in cellular metabolism, and they serve as intermediate metabolites affecting the biosynthesis of lipids, glutathione, nucleotides, glucosamine, and polyamines as well as cell proliferation and tricarboxylic acid circulating carbons, among other individual roles.^63,74^ In addition to the results exposed, the complementary blood chemistry shown in Table 3 suggested hepatic impairments as a consequence of the intake of the different diets. Hepatic complementary studies are needed to comprehensively understand these different protein-source related metabolomic signatures since the liver is an important organ for amino acid metabolism, which plays an important role in liver diseases.^74^

**Table 3:** Biochemical Profiling of the Blood of Experimental Rats after 10 Weeks of *Ad Libitum* Intake of the Different Experimental Diets (Beef (B), Commercial Seitan (S), or Commercial Tofu (T))

parameters^a^	B	S	T	
*p* value^b^				
TP (g/dL)	4.01 ± 0.74	4.80 ± 1.10	4.30 ± 0.73	ns
ALB (g/dL)	2.56 ± 0.40	2.89 ± 0.52	2.73 ± 0.38	ns
CREAT (mg/dL)	0.50 ± 0.12	0.49 ± 0.08	0.44 ± 0.11	ns
urea (mg/dL)	48.94ab ± 13.55	69.10a ± 21.52	36.50b ± 5.96	*
phosphorus (mg/dL)	3.46 ± 0.77	3.84 ± 0.77	3.93 ± 0.85	ns
TG (mg/dL)	125.14a ± 19.47	83.29b ± 26.49	74.86b ± 24.08	*
cholesterol (mg/dL)	76.29 ± 26.06	84.10 ± 20.72	72.08 ± 22.96	ns
ALP (U/l)	3.57b ± 1.02	6.29a ± 1.69	4.06b ± 1.10	*
ALT (U/l)	14.71b ± 3.99	28.53a ± 5.24	15.57b ± 6.79	*
GOT (U/l)	32.51b ± 8.63	46.71a ± 12.53	38.29b ± 9.12	*
HDL (mg/dL)	72.722 ± 17.17	86.17 ± 13.29	68.30 ± 16.34	ns
LDL (mg/dL)	8.71ab ± 1.11	9.29a ± 2.05	7.28b ± 1.70	*
GLB	1.26b ± 0.20	2.05a ± 0.51	1.37b ± 0.24	*

a Total protein content (TP), albumin (ALB), creatinine (CREAT), triglycerides (TG), alkaline phosphatase (ALP), alanine transaminase (ALT), aspartate transaminase (GOT), globulins (GLB).

b Significance level in the ANOVA test with the effects of diets (B, F, and F + P). *p < 0.05; **p < 0.01; ***p < 0.001; ns: not significant. Means with different letters within the same row were significantly different in the Tukey post hoc analysis (*p* < 0.05).

#### 3.2.4 Metabolism of Creatine and Carnitine

Certain discriminating compounds are known to be common constituents of the metabolomic profiling of fluids (blood/urine) upon intake of certain foods and hence may be used as biomarkers of diet/dietary patterns. This is the case of creatine, a common component of skeletal muscle, and its main degradation product, creatinine, which are elevated as a result of the intake of red meat.^75^ The significant differences in the urine concentrations of both creatine and creatinine (*p* < 0.001; FDR-adjusted *p* < 0.01 and *p* < 0.01; FDR-adjusted *p* < 0.05, respectively) between the dietary treatments (Figure 4) are consistent with previous studies in experimental animals and humans.^75,76^

Carnitine, another common component of skeletal muscle and a nutrient of growing interest, is also commonly found in red meat.^77^ Several authors established a link between intestinal microbiota, increased red meat consumption, and cardiovascular disease risk.^78–80^ The researchers hypothesized that certain gut microorganisms promoted the formation and intestinal uptake of the proatherogenic metabolite trimethylamine *N*-oxide (TMAO) when increased amounts of carnitine-rich foods were consumed. A higher abundance of TMAO in the blood of animals fed the B diet was found (*p* < 0.05; FDR-adjusted *p* < 0.05), which may be consistent with the higher amounts of carnitine previously shown in the gastrointestinal contents of B animals.^15^ TMAO is a gut-microbiota-dependent metabolite, and its concentration in the blood increases after consuming dietary L-carnitine, abundant in red meat, as long as the bacteria involved in TMAO formation are present in the gut.^79^ Vegans and vegetarians produce less TMAO from dietary L-carnitine compared to omnivorous diets, since chronic exposure to carnitine in subjects that regularly consume red meat would promote the growth of microorganisms better suited for TMAO production.^78^ Koeth et al.^78^ confirmed the crucial role of gut microbiota in TMAO production from dietary L-carnitine in mice but failed to identify the common genera of TMAO-producing microorganisms shared between humans and the murine model. The authors established a positive correlation between plasma levels of TMAO and several genera of bacteria such as *Prevotella*, *Alistipes*, *Lactobacillus*, and *Anaeroplasma*. Meanwhile, a negative correlation among plasma TMAO and *Bacteroides* spp. and *Parabacteroides* spp. was exposed in the study, together with unclassified *Erysipelotrichaceae* spp., *Turicibacter* spp., and unclassified *Desulfovibrionaceae* spp. Overall, these results agree with our metagenomic data and would explain the increased amounts of TMAO in the plasma of animals that consumed beef compared with those having the PBMAs. So, more insights are needed to establish direct associations between plasma TMAO, microbiota, red meat, and CVD risk, since TMAO also occurred in blood from animals exposed to the PBMAs, despite the low amounts of carnitine found in their gastrointestinal lumen.^15^

#### 3.2.5 Metabolism of Purines and Pyrimidines

Purines and pyrimidines are pivotal constituents of nucleic acids, and they also play a role as intermediate metabolites of lipid and carbohydrate metabolism.^81^ Purines may originate endogenously from the body or be externally provided by foods such as red meat and also plant-based foods.^82^ However, scientific literature is particularly profuse in identifying red meat as a dietary source of purines that contribute to the risk of several health disorders such gout and renal failure, which are usually characterized by hyperuricemia in the early stages.^82^

Uric acid (UA) is the end-product of purine degradation pathway, and it is mainly derived from endogenous synthesis.^82,83^ The occurrence of circulating UA depends on the balance between purine ingestion, *de novo* synthesis in cells, recycling, and the degradation function of xanthine oxidoreductase (XDH/XO) at the distal end of the purine pathway.^84^ Xanthine and hypoxanthine are intermediate metabolites of the purine degradation pathway: hypoxanthine is converted to xanthine through the activity of xanthine oxidase XO, and xanthine is eventually degraded to UA.^83^ It is highly remarkable that S animals were characterized by xanthinuria and hypoxanthinuria (*p* < 0.001; FDR-adjusted *p* < 0.01 in both cases). Yet, the abundances of UA were similar in the fluids from all animals irrespective of the experimental diets (*p* > 0.05). The intake of dietary purines (i.e., upon red meat intake) is commonly manifested as elevated amounts of UA in plasma and urine.^81^ On the same line, it is unusual that intermediate purine metabolites remain elevated in the blood/urine, and they may be indicating enzymatic disturbances affecting purine catabolic processes.^81^ Our data strongly suggest that rats fed on S had difficulties in metabolizing purines and pyrimidines because along with the aforementioned xanthinuria and hypoxanthinuria, the biological samples from S rats had elevated concentrations of pyrimidines as cytosine (blood) and uracil (urine). XO activity in rats is lower than that in humans, since xanthine is usually degraded to allantoin.^84^ Exploring allantoin and allantoin-related metabolites in both blood and urine, surprisingly, we found a compound matched as 3,5-dihydro-4H-pyrazolo­[3,4-*d*]­pyridin-4-one only in the urine of S animals (*p* < 0.001; FDR-adjusted *p* < 0.001) (Figure 4). The metabolite structure and molecular weight (Table S3) is closely related to allopurinol (1,5-dihydro-4H-pyrazolo­[3,4-*d*]­pyridin-4-one), a drug used in the treatment of hyperuricemia that inhibits XO activity in intestinal and hepatic tissues, where the enzyme is predominantly active, among others, with lower XO activity.^85,86^ Interestingly, allopurinol is also used in wheat plants to prevent a leaf infection called rust disease.^87^ It is known that allopurinol-treated wheat plants display disturbances in the metabolism of purines owing to the inhibition of XO.^88^ The occurrence of this potential inhibitor of XO activity in S rats and its connection with the impaired purine metabolism remain indefinite, but further investigation is worth doing. Increased levels of xanthine and hypoxanthine in urine should be regarded as a sign of health concern as they are commonly linked to renal disorders such as urolithiasis, which occurs in hereditary xanthinuria.^85^

#### 3.2.6 Other Discriminating Metabolites

Among the metabolites with significantly different abundances between the groups analyzed (i.e., ″discriminating metabolites”), some are associated with gut microbial processes and could be considered hallmarks of the use made by the microbiota of the different dietary protein sources under study. As an example, equol, a phytoestrogen, is produced by gut microbiota as a byproduct of metabolism of isoflavones, found in high concentrations in soy-based foods.^89^ So, it was no surprise that the levels of equol were the highest in both the blood and urine of the experimental animals that consumed the soy-derived diet (T; *p* < 0.001; FDR-adjusted *p* < 0.01). Equol is a bioactive metabolite with beneficial properties, such as antitumor qualities, associated, among others, with microorganisms from the family Eggerthellaceae.^89^ Increased abundance of uncultured bacterium from *Gordonibacter* spp. (Eggerthellaceae family) in the microbiota of T-animals (*p* < 0.05) might be related to the biosynthesis of the metabolite, but increased amounts of this microorganism were also shown in the microbiota of S-animals (*p* < 0.05). Anyway, the potential benefits of the long-term consumption of soy-based diet would be overshadowed by the previously mentioned growth of undesirable bacteria in T-microbiota and the presence of harmful metabolites in the metabolome of T rats, already explained in previous sections.

### 3.3 PBMA as Healthy Alternatives to Beef

Animal proteins have been largely criticized for their environmental footprint and health outcomes along the years, while plant proteins have been extensively praised in the scientific literature for its suitability and healthiness. Yet, the impact of ultraprocessing on the protein structure and composition and the effect of those changes on the nutritional value and health outcomes have been scarcely studied. This is of the utmost interest since vegan UPFs consumed in Western diets are considered a healthy replacement for animal sources of protein. As we previously reported, ultraprocessing seemed to affect the oxidative stability of PBMA proteins, which determines, in turn, the digestion of proteins in the stomach and jejunum and led to the fermentation of the undigested proteins by a shifted microbiota.^15^ This different digestion pattern may modulate the metabolism of nitrogen-containing substances by both the host and the microbiota, leading to different metabolic profiling in blood and urine, as explained as follows (Figure 5). According to multivariate analysis, different clustering of metabolites is shown in the PCA plots according to each type of fluid (Figure 5A, B: plasma and urine, respectively) and the different source of proteins supplied (B, S, or T), revealing that different sources of dietary proteins (animals vs plant) lead to clearly different blood and urine metabolomic fingerprints in Wistar rats. Although several metabolites with beneficial bioactivities were found in the fluids of PBMA animals, such as several indoles or equol, the presence of harmful bacteria taxa in their microbiota and the higher amounts of several hazardous metabolites in both their blood and urine, such as *p*-cresol or skatole, may lead to overall negative health outcomes as observed in gut health,^15^ markers of oxidative stress and liver function. Compared to blood from B rats, blood of S rats had increased amounts of some oxidative stress markers (i.e., total PPCs, pentosidine, and TBARs; *p* < 0.001), while T blood showed increased concentrations of both total PPCs and TBARs (*p* < 0.001). The increased gut oxidative stress, suggested previously when Wistar rats consumed PBMAs for 10 weeks,^15^ appeared to increase the oxidative status of the blood from PBMA animals. Additionally, the biochemical profile of the plasma, highlighted in Table 3, would support the hypothesized shift in the nitroso compound metabolism due to the intake of PBMAs, which could have resulted in lower amounts of amino acids being delivered to both the microbiota and the host as a consequence of the differential protein load and status. The alternative to the uptake of dietary amino acids for protein synthesis is catabolism, for which the end product is urea formation,^69^ and blood concentration of urea has been negatively correlated with protein quality by several authors.^90^ The highest amount of urea in the blood of S animals could be a hallmark of the defective amino acid metabolism hypothesized based on the results previously exposed. In regard to the concerns about damage of circulating increased oxidative stress and dangerous metabolites such as *p*-cresol or skatole to health, the increased amounts of plasmatic liver injury markers showed in S blood make these issues remarkable (Table 3). Hepatic energy and lipid metabolism, among many other metabolic pathways, seemed to be altered by the consumption of PBMAs. Additionally, inflammatory and immune responses appeared to be the mechanisms through which PBMAs as UPFs could transform the beneficial effects attributed to plant-diet consumption into harmful ones compared to beef (unpublished data).

**Figure 5:**
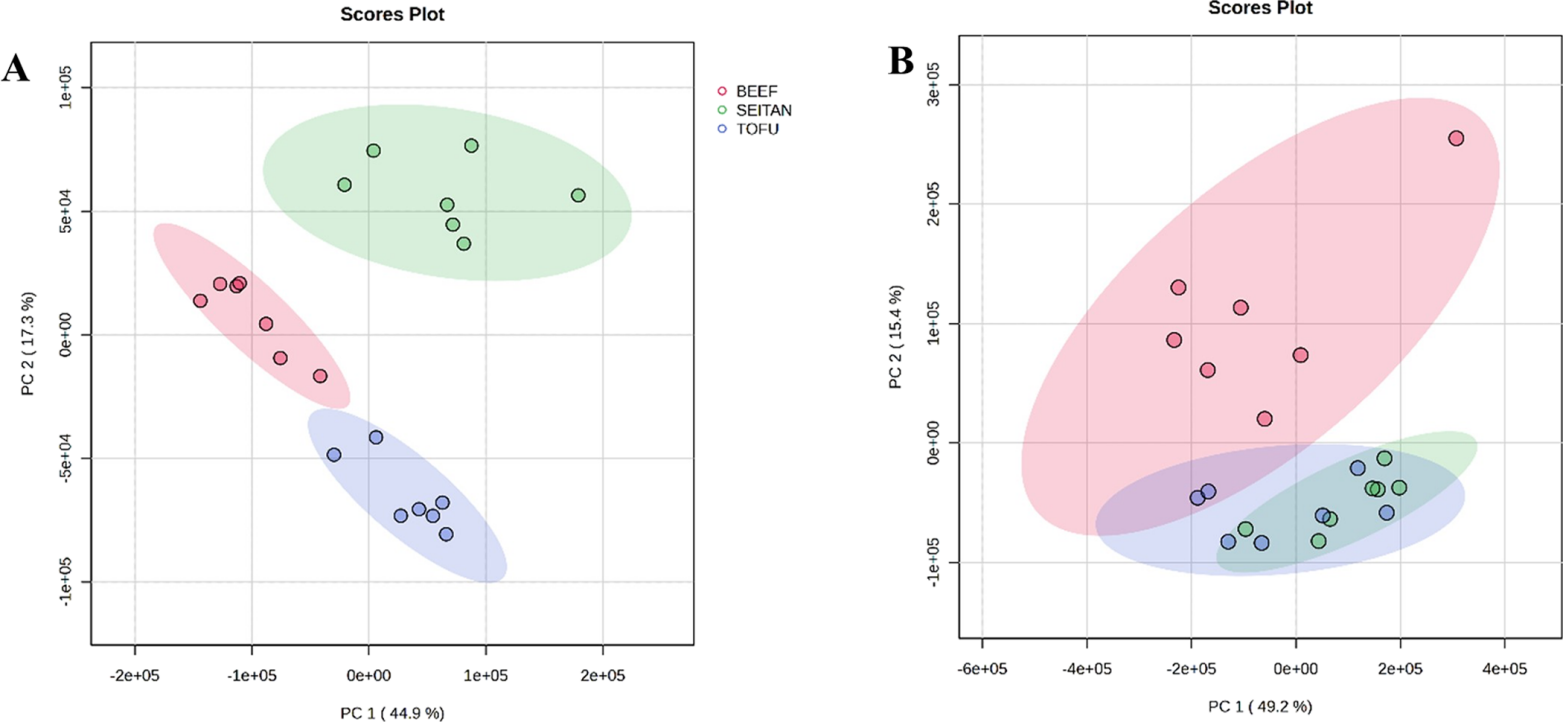
Score plots from the principal component analysis (PCA) multivariate analysis of both plasma (A) and urine (B) from Wistar rats (*n* = 7 per group) fed *ad libitum* for 10 weeks with a control base diet (based on beef) (B) and either PBMA-based diet (S and T, referred to seitan and tofu respectively).

In conclusion, the present results provide original insights into a report from the WHO (2021) in which the sustained intake of plant-based UPFs was linked to impaired physiological responses and chronic diseases. While unprocessed or minimally processed plant-based foods may deliver health promoting components (i.e., fiber, phytochemicals, vitamins, minerals), plant-derived UPF consumption seems to not only be deprived of beneficial effects but actually leads to metabolic disturbances driven by microbiota. According to what we know, our study provides relevant insights revealing that processing seriously affects protein oxidative stability, digestibility, and bioactivity, leading to undesirable microbiota changes and potential negative health outcomes. The proliferation of undesirable bacteria, the occurrence of harmful metabolites such as *p*-cresol or skatole in blood and urine, and metabolic impairment of amino acids, purines, and pyrimidines were among the consequences of a long-term consumption of UPF based on plant proteins. The adverse effects associated with an excessive intake of oxidized proteins such as that from PBMAs may overshadow the benefits provided by the consumption of plant-based foods, promoting an unhealthy scenario available to the development of several diseases. Further studies would overcome some of the limitations of the present study including (i) extending the exposure to vegan UPFs for a longer time and specifically assessing the implication of these foods in chronic diseases such as obesity and T2DM, (ii) dedicated investigations to quantify and unveil the pathophysiological mechanisms of mutagenic metabolites found in colon, blood, and urine of rats fed the vegan UPFs (i.e., *p*-cresol), and (iii) confirming some of the speculative negative health outcomes herewith hypothesize in human volunteers replacing ABFs by these vegan UPFs.

## Supplementary Material





## Notes

Detailed information on the ingredients and nutritional composition of the PBMAs used to produce the different feeds (Table S1), composition and caloric value of experimental feeds based on the different sources of protein (Table S2), and metabolite characterization (Table S3) (PDF)
Relative abundances of the genus (FRL6) and species (FRL7) of microorganisms, ANOVA results from statistical analysis of plasma (ANOVA_Plasma), and urine (ANOVA_Urine) metabolite intensities (XLSX)

## Abbreviations

PBMAs: plant-based meat analogues
UPFs: ultraprocessed foods
CRC: colorectal cancer
ABF: animal-based foods
MA: meat analogues
VM: vegan meat
T2DM: type 2 diabetes mellitus
CVD: cardiovascular diseases
WHO: World Health Organization
GIT: gastrointestinal tract
α-AS: α-aminoadipic semialdehyde
γ-GS: γ-glutamic semialdehyde
PPC: primary protein carbonyls
APOPs: advanced protein oxidation products
FU: fluorescent units
TBARs: thiobarbituric acid reactive substances
MDA: malondialdehyde
BHT: butylated hydroxytoluene
TEP: 1,1,3,3-tetraethoxypropane
TBA: thiobarbituric acid
TP: total protein content
ALB: albumin
GLB: globulins
CREAT: creatinine
ALP: alkaline phosphatase
ALT: alanine transaminase
AST/GOT: aspartate transaminase/glutamic oxaloacetic transaminase
TG: triglycerides
OTUs: operational taxonomic units
PCA: principal component analysis
SRB: sulfate-reducing bacteria
IBD: intestinal bowel diseases
H2S: hydrogen sulfide
ROS: reactive oxygen species
Tyr: tyrosine
Trp: tryptophan
IAA: indole-3-acetic acid
IPA: indole-3-propionic acid
IALD: indole-3-aldehyde
IAALD: indole-3-acetaldehyde
Lys: lysine
Val: valine
Met: methionine
Ala: alanine
IAA: indispensable amino acid
GABA: γ-aminobutyric acid
BCAA: branched-chain amino acids
TMAO: trimethylamine N-oxide
UA: uric acid
XDH/XO: xanthine oxidoreductase
