# Supplementary material for: Deciphering the Physiological Responses to the Intake of Plant-Based Meat Analogues: On the Track of Microbiota and Biomarkers in Serum and Urine
Source: J Agric Food Chem. 2025 Jul 17;73(36):22698–713. doi: 10.1021/acs.jafc.5c02799 (PMC12426926; doi:10.1021/acs.jafc.5c02799)
Supplement: Supplementary file 1 [file jf5c02799_si_001.pdf]

Supporting Information

Deciphering the Physiological Responses to the Intake of Plant-Based Meat Analogues: On the Track of Microbiota and Biomarkers in Serum and Urine

Guadalupe Sánchez-Terrón[1*], Remigio Martínez[2], David Morcuende[1] & Mario Estévez[1]

[1] TECAL Research Group, Meat and meat products research institute (IPROCAR), Universidad de Extremadura (UEX), Cáceres, 10003, Spain

[2] Animal Health Department, Animal Health and Zoonoses Research Group (GISAZ), UIC Zoonosis and Emergent Diseases (ENZOEM Competitive Research Unit), Universidad of Córdoba (UCO, ROR-ID 05yc77b46), Córdoba, 14014, Spain

* Correspondence: masanchezt@unex.es

Contents of SI:

TABLE S1. Detailed information of ingredients and nutritional composition of commercial seitan and tofu used to make the different feeds based on plant sources supplied to the animals during the experiment..2

TABLE S2. Composition and caloric value of experimental feeds based on either animal-protein diet (B), or plant-based protein diets, i.e.: seitan diet (S) and tofu diet (T) (means ± standard deviation)..3

TABLE S3. Metabolites characterization...4

TABLE S1. Detailed information of ingredients and nutritional composition of commercial seitan and tofu used to make the different feeds based on plant sources supplied to the animals during the experiment.

Commercial "Seitán" (Hacendado)		***Commercial "Tofu firme" (Hacendado)***	
**Composition:** Water, wheat flour, wheat gluten, *shoyu* soy sauce (water, soybeans, wheat, sea salt, koji mold), *tamari* soy sauce (water, soybeans, sea salt, *mikawa* fermented rice and koji mold), kombu seaweed, ginger, garlic.		**Composition:** Water, soybeans, marine magnesium chloride (nigari), calcium chloride.	
**Nutritional information (per 100g)**		**Nutritional information (per 100g)**	
Energy	525 kJ/124 kcal	Energy	459 kJ/110 kcal
Total fat	1.8g	Total fat	6.9g
Saturated fat	0.5g	Saturated fat	1.2g
Total carbohydrates	2.9g	Total carbohydrates	0.9g
Sugars	0.4g	Sugars	< 0.5g
Dietary fiber	0.1g	Dietary fiber	< 0.5g
Sodium	0.58g	Sodium	0.04g

TABLE S2. Composition and caloric value of experimental feeds based on either animal-protein diet (B), or plant-based protein diets, i.e.: seitan diet (S) and tofu diet (T) (means ± standard deviation).

	B	S	T	*p* value[1]
Protein[2]	30.41±1.26	30.15±0.99	29.35±1.04	ns
Moisture[2]	6.88±0.25	6.99±0.85	6.47±0.15	ns
Carbohydrates[2]	55.80ab±2.12	58.20a±3.01	53.70b±1.98	*
Lipids[2]	4.20b±0.33	2.35c±0.64	8.40a±0.36	***
SFA[3]	28.86a±1.64	17.97b±1.27	15.73b±1.15	**
MUFA[3]	31.52a±1.58	20.60b±1.31	22.62b±1.20	**
PUFA[3]	39.61b±1.61	61.43a±1.12	61.65a±1.15	***
Ashes[2]	3.20a±0.18	2.28b±0.53	2.18b±0.40	*
Caloric value[4]	3.92±0.38	3.89±0.33	4.02±0.36	ns

[1] Significance level in ANOVA; *: p<0.05; **: p<0.01; ***: p<0.001; ns: no significant. Means with different letters within the same row were significantly different in Tukey post-hoc analyses.

[2] Results expressed as g/100 g feed.

[3] SFA: Saturated fatty acids; MUFA: Monounsaturated fatty acids; PUFA: Polyunsaturated fatty acids. Results expressed in percentage.

[4] Results expressed in Kcal/g feed.

TABLE S3. Metabolites characterization.

Name	Fluid	Formula	Structure	Cal. MW	m/z	MS2	Reference ion	SMILES	InChi
1-Piperideine	Blood	C5 H9 N	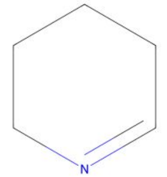	83.073	84.080	No MS2	[M+H]+1	C1CCN=CC1	InChI=1S/C5H9N/c1-2-4-6-5-3-1/h4H,1-3,5H2
3,5-Dihydro-4H-pyrazolo[3,4-d]pyridine-4-one	Urine	C5 H4 N4 O	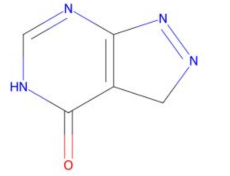	136.038	137.045			c1[nH]c(=O)c2c(n1)N=NC2	InChI=1S/C5H4N4O/c10-5-3-1-8-9-4(3)6-2-7-5/h2H,1H2,(H,6,7,10)
3-Carboxyindole	Blood	C9 H7 N O2	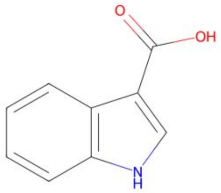	161.047	162.054	No MS2	[M+H]+1	c1ccc2c(c1)c(c[nH]2)C(=O)O	InChI=1S/C9H7NO2/c11-9(12)7-5-10-8-4-2-1-3-6(7)8/h1-5,10H,(H,11,12)
Alanine	Blood Urine	C3 H7 N O2	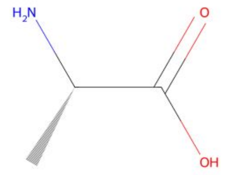	89.047	90.054	No MS2	[M+H]+1	C[C@@H](C(=O)O)N	InChI=1S/C3H7NO2/c1-2(4)3(5)6/h2H,4H2,1H3,(H,5,6)/t2-/m0/s1

TABLE S3. Continued.

Name	Fluid	Formula	Structure	Cal. MW	m/z	MS2	Reference ion	SMILES	InChi
Creatine	Urine	C4 H9 N3 O2	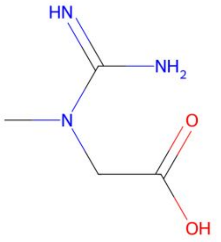	131.069	132.076	DDA for preferred ion	[M+H]+1	CN(CC(=O)O)C(=N)N	InChI=1S/C4H9N3O2/c1-7(4(5)6)2-3(8)9/h2H2,1H3,(H3,5,6)(H,8,9)
Creatinine	Urine	C4 H7 N3 O	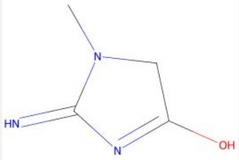	113.058	114.066	DDA for preferred ion	[M+H]+1	CN1CC(=NC1=N)O	InChI=1S/C4H7N3O/c1-7-2-3(8)6-4(7)5/h2H2,1H3,(H2,5,6,8)
Cysteine	Urine	C3 H7 N O2 S	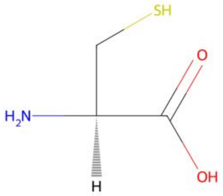	121.019	122.026	DDA for preferred ion	[M+H]+1	C([C@@H](C(=O)O)N)S	InChI=1S/C3H7NO2S/c4-2(1-7)3(5)6/h2,7H,1,4H2,(H,5,6)/t2-/m0/s1
Cytosine	Blood	C4 H5 N3 O	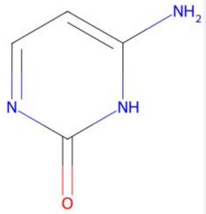	111.043	112.050	DDA for preferred ion	[M+H]+1	c1cnc(=O)[nH]c1N	InChI=1S/C4H5N3O/c5-3-1-2-6-4(8)7-3/h1-2H,(H3,5,6,7,8)

TABLE S3. Continued.

Name	Fluid	Formula	Structure	Cal. MW	m/z	MS2	Reference ion	SMILES	InChi
Equol	Blood Urine	C15 H14 03	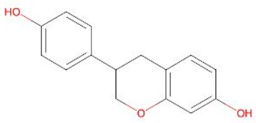	242.094	243.101	DDA for preferred ion	[M+H]+1	c1cc(ccc1C2Cc3ccc(cc3OC2)O)O	InChI=1S/C15H14O3/c16-13-4-1-10(2-5-13)12-7-11-3-6-14(17)8-15(11)18-9-12/h1-6,8,12,16-17H,7,9H2
Fructoselysine	Urine	C12 H24 N2 O7	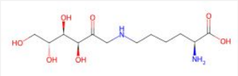	308.157	309.165	DDA for preferred ion	[M+H]+1	C(CCNCC(=O)[C@H]([C@@H]([C@@H](CO)O)O)O)C[C@@H](C(=O)O)N	InChI=1S/C12H24N2O7/c13-7(12(20)21)3-1-2-4-14-5-8(16)10(18)11(19)9(17)6-15/h7,9-11,14-15,17-19H,1-6,13H2,(H,20,21)/t7-,9+,10+,11+/m0/s1
Glutamic acid	Urine	C5 H9 N O4	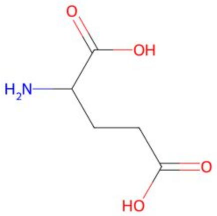	147.052	148.060	DDA for other ion	[M+H]+1	C(CC(=O)O)C(C(=O)O)N	InChI=1S/C5H9NO4/c6-3(5(9)10)1-2-4(7)8/h3H,1-2,6H2,(H,7,8)(H,9,10)
Histidine	Urine	C6 H9 N3 O2	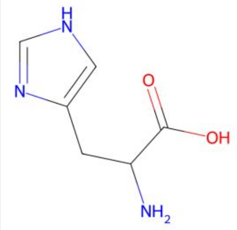	155.069	156.076	No MS2	[M+H]+1	c1c(nc[nH]1)CC(C(=O)O)N	InChI=1S/C6H9N3O2/c7-5(6(10)11)1-4-2-8-3-9-4/h2-3,5H,1,7H2,(H,8,9)(H,10,11)

TABLE S3. Continued.

Name	Fluid	Formula	Structure	Cal. MW	m/z	MS2	Reference ion	SMILES	InChi
Hypoxanthine	Urine	C5 H4 N4 O	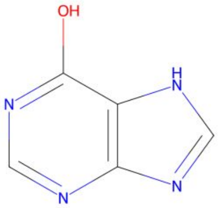	136.038	137.045	DDA for other ion	[M+H]+1	c1[nH]c2c(n1)ncnc2O	InChI=1S/C5H4N4O/c10-5-3-4(7-1-6-3)8-2-9-5/h1-2H,(H2,6,7,8,9,10)
Indole	Blood<br>Urine	C8 H7 N	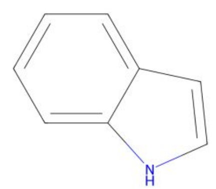	117.057	118.064	No MS2	[M+H]+1	c1ccc2c(c1)cc[nH]2	InChI=1S/C8H7N/c1-2-4-8-7(3-1)5-6-9-8/h1-6,9H
Indole-3-acetic acid	Blood	C10 H9 N O2	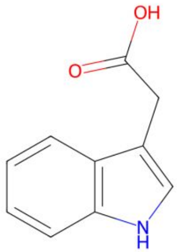	175.063	176.070	No MS2	[M+H]+1	c1ccc2c(c1)c(c[nH]2)CC(=O)O	InChI=1S/C10H9NO2/c12-10(13)5-7-6-11-9-4-2-1-3-8(7)9/h1-4,6,11H,5H2,(H,12,13)
Lysine	Blood<br>Urine	C6 H14 N2 O2	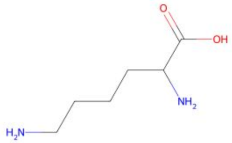	146.105	147.112	DDA for preferred ion	[M+H]+1	C(CCN)CC(C(=O)O)N	InChI=1S/C6H14N2O2/c7-4-2-1-3-5(8)6(9)10/h5H,1-4,7-8H2,(H,9,10)

TABLE S3. Continued.

Name	Fluid	Formula	Structure	Cal. MW	m/z	MS2	Reference ion	SMILES	InChi
Methionine	Blood	C5 H11 N O2 S	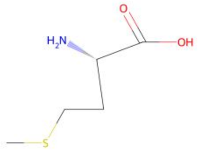	149.050	150.058	DDA for preferred ion	[M+H]+1	CSCC[C@@H](C(=O)O)N	InChI=1S/C5H11NO2S/c1-9-3-2-4(6)5(7)8/h4H,2-3,6H2,1H3,(H,7,8)/t4-/m0/s1
N6-Methyladenosine	Urine	C11 H15 N5 O4	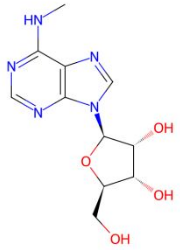	281.111	282.119	DDA for preferred ion	[M+H]+1	CNc1c2c(ncn1)n(cn2)[C@H]3[C@@H]([C@@H]([C@H](O3)CO)O)O	InChI=1S/C11H15N5O4/c1-12-9-6-10(14-3-13-9)16(4-15-6)11-8(19)7(18)5(2-17)20-11/h3-5,7-8,11,17-19H,2H2,1H3,(H,12,13,14)/t5-,7-,8-,11-/m1/s1
Norvaline	Blood	C5 H11 N O2	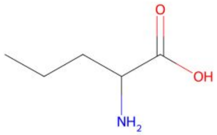	117.07	118.08	DDA for other ion	[M+H]+1	CCCC(C(=O)O)N	InChI=1S/C5H11NO2/c1-2-3-4(6)5(7)8/h4H,2-3,6H2,1H3,(H,7,8)
p-Cresol	Blood Urine	C7 H8 O	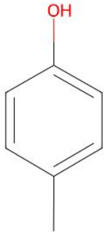	108.057	109.064	No MS2	[M+H]+1	Cc1ccc(cc1)O	InChI=1S/C7H8O/c1-6-2-4-7(8)5-3-6/h2-5,8H,1H3

TABLE S3. Continued.

Name	Fluid	Formula	Structure	Cal. MW	m/z	MS2	Reference ion	SMILES	InChi
Phenol	Blood	C6 H6 O	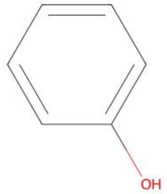	94.041	95.049	No MS2	[M+H]+1	c1ccc(cc1)O	InChI=1S/C6H6O/c7-6-4-2-1-3-5-6/h1-5,7H
Skatole	Urine	C9 H9 N	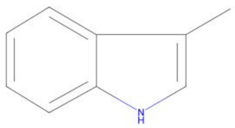	131.073	132.080	DDA for preferred ion	[M+H]+1	Cc1c[nH]c2c1cccc2	InChI=1S/C9H9N/c1-7-6-10-9-5-3-2-4-8(7)9/h2-6,10H,1H3
Threonine	Urine	C4 H9 N O3	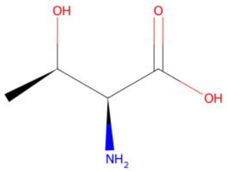	119.058	120.065	DDA for preferred ion	[M+H]+1	C[C@H]([C@@H](C(=O)O)N)	InChI=1S/C4H9NO3/c1-2(6)3(5)4(7)8/h2-3,6H,5H2,1H3,(H,7,8)/t2-,3+/m1/s1
Trimethylamine N-oxide	Blood	C3 H9 N O	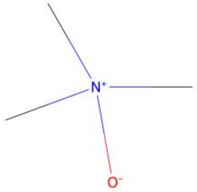	75.068	76.075	No MS2	[M+H]+1	C[N+](C)(C)[O-]	InChI=1S/C3H9NO/c1-4(2,3)5/h1-3H3
Tryptophan	Blood	C11 H12 N2 O2	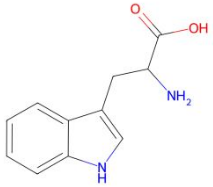	204.089	205.096	No MS2	[M+H]+1	c1ccc2c(c1)c(c[nH]2)CC(C(=O)O)N	InChI=1S/C11H12N2O2/c12-9(11(14)15)5-7-6-13-10-4-2-1-3-8(7)10/h1-4,6,9,13H,5,12H2,(H,14,15)

TABLE S3. Continued.

Name	Fluid	Formula	Structure	Cal. MW	m/z	MS2	Reference ion	SMILES	InChi
Tryptophol	Urine	C10 H11 N O	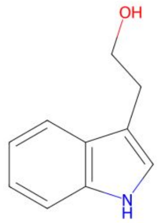	161.083	162.091	DDA for preferred ion	[M+H]+1	c1ccc2c(c1)c(c[nH]2)CCO	InChI=1S/C10H11NO/c12-6-5-8-7-11-10-4-2-1-3-9(8)10/h1-4,7,11-12H,5-6H2
Tyrosine	Blood	C9 H11 N O3	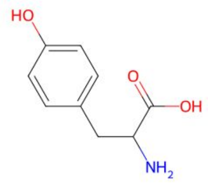	181.07	182.08	DDA for other ion	[M+H]+1	c1cc(ccc1CC(C(=O)O)N)O	InChI=1S/C9H11NO3/c10-8(9(12)13)5-6-1-3-7(11)4-2-6/h1-4,8,11H,5,10H2,(H,12,13)
Uracil	Urine	C4 H4 N2 O2	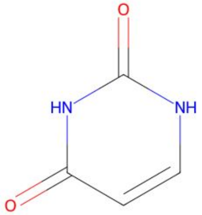	112.027	112.027	No MS2	[M+H]+1	c1c[nH]c(=O)[nH]c1=O	InChI=1S/C4H4N2O2/c7-3-1-2-5-4(8)6-3/h1-2H,(H2,5,6,7,8)
Valine	Blood	C5 H11 N O2	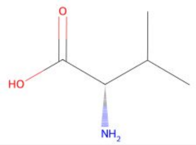	117.078	118.086	No MS2	[M+H]+1	CC(C)[C@@H](C(=O)O)N	InChI=1S/C5H11NO2/c1-3(2)4(6)5(7)8/h3-4H,6H2,1-2H3,(H,7,8)/t4-/m0/s1
Xanthine	Blood Urine	C5 H4 N4 O2	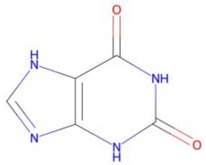	152.033	153.040	DDA for preferred ion	[M+H]+1	c1[nH]c2c(n1)[nH]c(=O)[nH]c2=O	InChI=1S/C5H4N4O2/c10-4-2-3(7-1-6-2)8-5(11)9-4/h1H,(H3,6,7,8,9,10,11)
